# Controlled release of coated antioxidants inhibits *Citrobacter rodentium* colonization in the colon of rats by reducing gut redox potential

**DOI:** 10.1016/j.redox.2026.104005

**Published:** 2026-01-04

**Authors:** Ni Feng, Changsong Fu, Jinwei You, Dongfang Wang, Xiaobo Feng, Yong Su

**Affiliations:** a Laboratory of Gastrointestinal Microbiology, Jiangsu Key Laboratory of Gastrointestinal Nutrition and Animal Health, College of Animal Science and Technology, Nanjing Agricultural University, Nanjing, China; b Department of General Surgery, Jinling Hospital, Affiliated Hospital of Medical School, Nanjing University, Nanjing, China; c Department of Laboratory Animal, Jinling Hospital, Affiliated Hospital of Medical School, Nanjing University, Nanjing, China

**Keywords:** Gut health, Redox potential, *C. rodentium*, Colonization, Controlled release

## Abstract

Intestinal redox potential serves as a critical parameter reflecting the dynamic characteristics of the gut microenvironment. To precisely modulate the intestinal redox potential and evaluate its inhibition of pathogenic colonization, this study built a controlled release system and further investigated its role in gut health under a lower redox potential. The results demonstrated that the controlled release formulation significantly reduced fecal redox potential more effectively than uncoated antioxidants. By optimizing the hydrodynamic size and zeta potential of ethoxyquin (EQ) and ferulic acid (FA), the coated FA formulation maintained high efficiency in reducing redox potential and reversed body weight loss induced by pathogenic infection. Both coated EQ (EQC) and FA (FAC) selectively enriched beneficial genera, such as *Lactobacillus* and *Limosilactobacillus*, while suppressing opportunistic pathogens like *Klebsiella*. Notably, coated FA demonstrated enhanced efficacy in alleviating *Citrobacter rodentium* (*C. rodentium*)-induced weight loss and reducing pathogens burden compared to uncoated FA. Mechanistically, coated FA promoted the enrichment of *Lactobacillus reuteri* (*L*. *reuteri)*, suppressed the proliferation of Enterobacteriaceae, and enhanced intestinal *Muc2* gene expression. Functional metagenomic analysis revealed that FAC significantly downregulated ABC transporter activity in Enterobacteriaceae, thereby impairing biofilm formation and synergizing with mucus secretion to inhibit pathogen colonization. Further *in vitro* co-culture trials confirmed that under a lower redox system, *L*. *reuteri* had a stronger inhibitory effect on *C. rodentium* as well as the expression of their virulence genes (*(tir, ler*). Collectively, these findings suggest that precise modulation of colonic redox potential through controlled release strategies represents a promising approach to enhance host defense against enteric pathogens via microbiota reprogramming.

## Graphical abstract

**Scheme: Graphical scheme of controlled release delivery and its enhancement of colonization resistance against Enterobacteriaceae through modulation of virulence and microbiota function.** Following oral administration, the coated antioxidants regulated gut redox potential, thereby reshaping the composition of microbiota in colon. The formulation effectively lowered redox potential, enriched beneficial microbiota (including *Akkermansia, Lactobacillus reuteri,* and *Limosilactobacillus*), and enhanced mucus secretion (↑*Muc2*), thereby strengthening the resistance to pathogens colonization. Mechanistically, this approach inhibited quorum sensing and biofilm formation in enteric pathogens by restricting iron availability and *Lactobacillus reuteri* suppressed *C. rodentium* growth and virulence under low redox potential.

**Figure:**
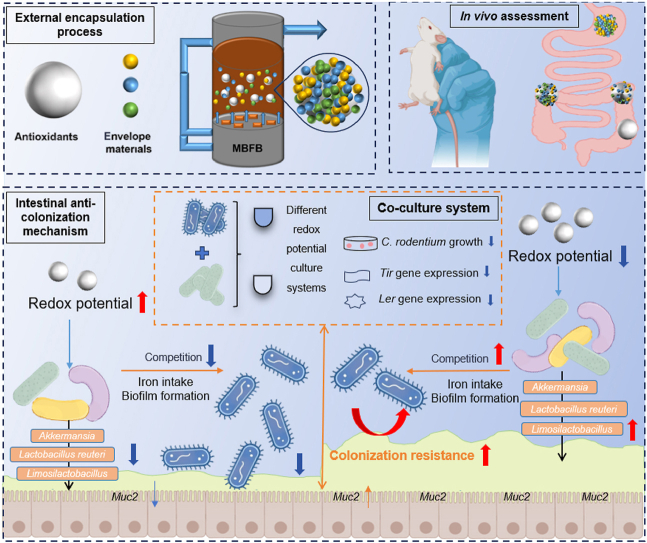

## 1 Introduction

Redox potential as one of the core physicochemical parameters of environmental microecology, profoundly influences microbial metabolic activity and intestinal health. Dietary supplementation with commonly used antioxidants encompasses both chemically synthesized and naturally derived varieties. In broiler models, EQ supplementation was demonstrated to enhance intestinal antioxidant capacity, alleviate oxidative stress, and potentially inhibit harmful bacteria while promoting the growth of beneficial bacteria [1]. Although synthetic antioxidant EQ is inexpensive, potent lipid-oxidation inhibitors with established production pipelines, they can bioaccumulate, contaminate the environment, disrupt endocrine function, and leave residues in animal products, which may undermine consumer acceptance [2]. By contrast, natural antioxidant FA exhibits anti-inflammatory, antibacterial, antiviral, and immunomodulatory properties. These characteristics align with green-farming initiatives and food-safety priorities. Mechanistic studies indicated FA-butyrate synergy elevated *Lactobacillus* abundance and suppresses pro-inflammatory cytokines to enhanced intestinal immune barrier function [3]. *In vitro* fermentation experiment confirmed that FA promoted short-chain fatty acids (SCFAs) production, particularly butyrate and propionate, which inhibited pathogenic bacteria growth and maintained intestinal pH balance [4]. Comparative analyses revealed that the natural antioxidants significantly reduced intestinal redox potential, decreased nutritional diarrhea incidence, and maintained intestinal health [5]. Nevertheless, challenges in extraction efficiency and production scalability limit natural antioxidant applications, necessitating development of targeted delivery systems. Fluidized bed spray coated technology enables fabrication of microsphere-granule composites that enhance colonic drug bioavailability through pH-responsive release kinetics [6]. This approach potentially optimizes antioxidant's therapeutic effects by protecting gastric labile components while ensuring microbial biotransformation in distal gut regions. However, it remains unclear whether coated antioxidants can better regulate intestinal redox potential through colon-targeted release and play benifit roles in colon health.

Gram-negative opportunistic pathogens such as *Citrobacter rodentium* deploy multi-tiered redox adaptation mechanisms during host colonization [7]. Studies have shown that this pathogen maintains metabolic activity across a broad redox potential range (−200 to **+**200 mV), granting it a competitive edge in dynamic host environments. *C. rodentium* dynamically modulates virulence gene (*tir* and *ler*) expression in response to intestinal oxygen tension, thereby attenuating pathogen virulence and mediates adhesion to intestinal epithelial cells [8]. This underscores the importance of maintaining a low intestinal redox potential to enhance colonization resistance. Redox potential serves as a master regulator of microbial energetics across intestinal, aquatic, and clinical ecosystems and survival mechanisms by regulating the activity of the electron transport chain [5,9]. Reducing the redox potential can enhance the surface adhesion capability of bacteria and potential redirect microbial metabolism towards reductive pathways [10]. Dietary fiber supplementation decreased the intestinal redox potential and stimulated the proliferation of anaerobic bacteria, including those in the Lachnospiraceae family [11]. These communities maintain a hypoxic environment via fermentation, establishing an ecological niche favorable to the colonization of obligate anaerobes. Simultaneously, a low redox potential suppresses excessive mucin degradation [12]. In contrast, a high redox potential compels microorganisms to degrade mucin for nutrients, leading to mucosal layer disruption and increased risk of inflammation [13]. A low redox potential enhances the growth of butyrate-producing bacteria, whose metabolites directly upregulate mucin synthesis genes [14], thereby reinforcing the integrity of the mucus barrier. We previously demonstrated that pectin supplementation coordinated with reduced intestinal redox potential and accelerated post-antibiotic reconstruction of gut microbiota [15]. While dietary fibers maintain low colonic redox potential through oxidative stress mitigation [16], antioxidant bioavailability emerges as a key modulator of intestinal redox potential. Altering the redox potential represents a viable strategy for the targeted modulation of the gut microbiota to enhance intestinal health.

Building upon these findings, the present study aimed to investigate whether reducing intestinal redox potential could attenuate pathogen colonization associated with infectious diarrhea. Specifically, we assessed the effects of uncoated antioxidants and coated antioxidants on redox potential and pathogen load, and elucidated the mechanisms through which colon-targeted systems modulate microbial colonization. A deeper understanding of these mechanisms will provide a theoretical basis for novel antimicrobial strategies targeting gut redox potential and offer valuable insights for developing innovative modulators of the intestinal microenvironment.

## 2 Materials and methods

### 2.1 Fluidized bed coated process

In our previous study, nine distinct classes of redox potential-regulating antioxidants were systematically screened using both *in vitro* and *in vivo* models, leading to the selection of EQ and FA for their superior efficacy [17]. A fluidized bed rotor granulator was employed as the core apparatus for the coated process. Gas-liquid-solid three-phase mixing was enhanced by optimizing the bottom multi-nozzle configuration, supported by computational fluid dynamics simulations to improve catalyst dispersion and mass transfer efficiency within the system [18]. The coated solution consisted of EQ and FA were ethylcellulose polymer, palm oil, and silica in a ratio of 2:2:1. A multi-layer coated strategy was applied to achieve controlled release. Uniform particle fluidization was maintained by controlling the inlet air temperature at 30 °C and the fluidizing gas velocity at 1 m/s, thereby preventing agglomeration and entrainment. The spray rate of the coated solution (8 mL/min) and the atomization pressure (1.5 bar) were carefully regulated to ensure uniform droplet distribution. A staged drying process was implemented: an initial low-temperature phase (40 °C) to prevent coated rupture, followed by a higher-temperature phase (60 °C) to accelerate solvent evaporation [19]. The contents of EQ and FA after coated were 24 % and 12 % respectively.

### 2.2 In vitro evaluation of coated antioxidants

#### 2.2.1 Hydrodynamic size and zeta potential measurement

**Fig. 1:**
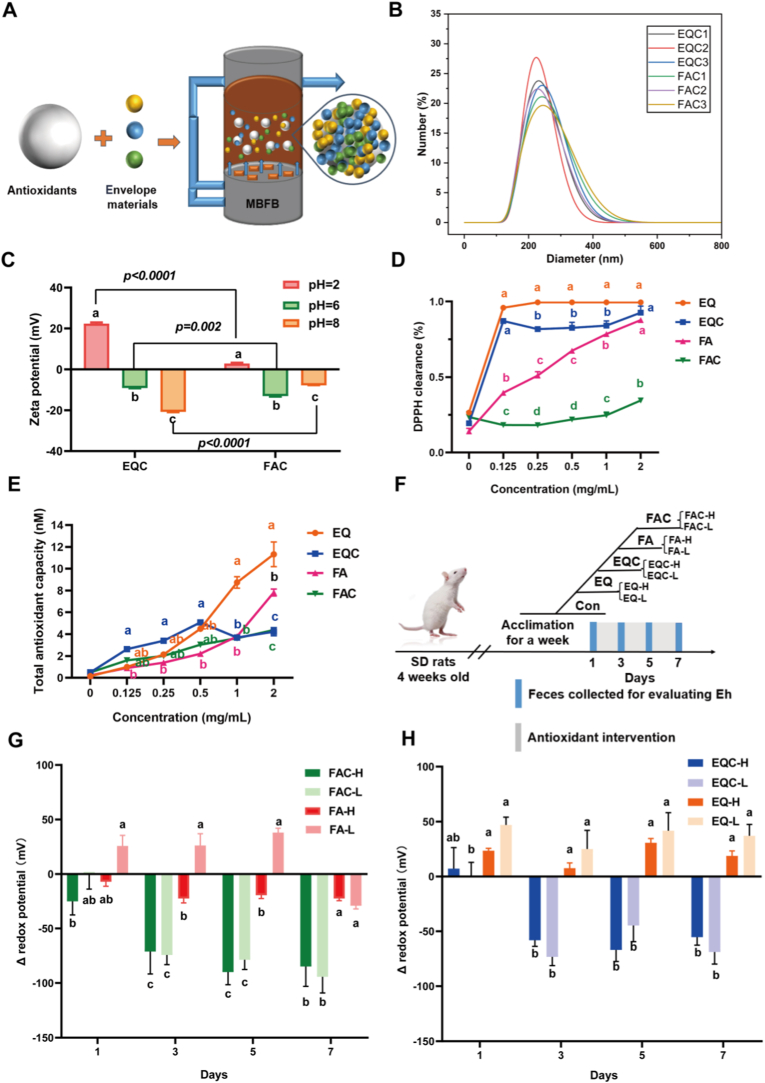
Coated EQ and FA regulated intestinal redox potential. (A) Flowchart of fluidized bed coated process. (B, C) Particle size and zeta potential of coated antioxidants. (D, E) DPPH activity and FRAP activity evaluation of coated and uncoated antioxidants. (F) *In vivo* intervention experiments and group design for coated antioxidants. n = 5 in per group. (G, H) Redox potential efficiency between coated and uncoated antioxidants. Different letters indicated significant differences (*P* < 0.05), while identical letters represented non-significant differences (*P* > 0.05).

The hydrodynamic diameter of coated materials was determined by analyzing fluctuations in scattered light caused by brownian motion of particles in solution (Fig. 1A). Aggregation of nanoparticles into micron-sized clusters during fluidized bed coated may hinder direct measurement of primary nanoparticle size via dynamic light scattering, necessitating ultrasonic dispersion or solvent treatment [20]. Surface charge modulation through pH adjustment was used to assess particle stability under simulated gastrointestinal conditions [21].

#### 2.2.2 Antioxidant activity determination of different antioxidants

The antioxidant capacities of EQ, EQC, FA, FAC were evaluated using 2,2-diphenyl-1-picrylhydrazyl (DPPH) [22] radical scavenging and ferric reducing antioxidant power (FRAP) [23] assays. DPPH scavenging capacity was determined by mixing 10 μL sample with 190 μL DPPH solution, followed by 30 min dark incubation. Absorbance at 515 nm was quantified using a microplate reader. For FRAP assay (Yeasen Biotechnology), 30 μL sample was reacted with 264 μL FRAP working solution at 37 °C for 30 min, and absorbance at 593 nm was measured. Antioxidant capacity was expressed as μmol Fe^2+^ equivalents/mL based on a standard curve. EQC maintaining 89.4 % of the DPPH radical scavenging capacity of native EQ, whereas FAC showed 62.3 % reduction compared to unmodified FA (Fig. 1D and F). Both coated antioxidants exhibited concentration-dependent FRAP activity.

### 2.3 In vivo evaluation of coated antioxidants

All animal experimental procedures were approved by Nanjing Agricultural University's Institutional Animal Care Committee (SYXK2019-0066), complying with China's experimental animal guidelines (EACUGC2018-01). Based on our previous study [17], a power analysis (α = 0.05, power >0.80) for detecting a medium effect size (Δ = 0.5) indicated that a minimum of six animals per group was required. Four-week-old male Sprague-Dawley (SD) rats (Cavens Biotechnology, Suzhou) underwent 7-day acclimation before randomization into five groups (n = 6): control (Con), low/high-concentration EQC (EQC-L/H) and EQ (EQ-L/H), low/high-concentration FAC (FAC-L/H) and FA (FA-L/H). Rats were individually housed in stainless steel cages with wire mesh bottoms under controlled temperature (22 ± 2 °C), humidity (50–60 %), and a 12 h light/dark cycle. Dietary formulations for coated and uncoated antioxidants were detailed in Table S1 and S2. Low and high concentration of antioxidants were incorporated into the basal diet of each corresponding group. Fecal samples were collected on days 1, 3, 5, and 7 of the intervention period for the determination of redox potential.

### 2.4 Experimental design for *C. rodentium* challenge

The *C. rodentium* strain used for the challenge experiment was obtained from our laboratory. Bacterial stocks were retrieved from a −80 °C freezer, inoculated into LB broth, and cultured overnight at 37 °C with shaking. The bacterial suspension was serially diluted and plated on LB solid medium supplemented with 50 mg/mL antibiotics, followed by 24-h incubation. A single colony was also cultured in LB liquid medium overnight. The overnight culture was subcultured in fresh LB medium at a 1:20 ratio for 6–8 h, and the OD_600_ was measured. The bacterial suspension was diluted to 0.12 OD/mL, and the challenge dose was 200 μL per rat. Two independent animal experiments were conducted to evaluate the effects of antioxidant coated formulations on *C. rodentium* colonization and gut microbiota modulation.

In Experiment 1, SD rats were acclimated for one week and randomly assigned to six groups (n = 6): control (Con), *C. rodentium*-challenged control (Con-Cr), low/high-concentration EQC with *C. rodentium* challenge (EQC-L-Cr, EQC-H-Cr), low/high-concentration FAC with *C. rodentium* challenge (FAC-L-Cr, FAC-H-Cr). Rats in all groups received a basal diet with *ad libitum* access to food and water. Encapsulation procedures and diet formulations are detailed in Table S1 and S2. *C. rodentium* challenge was administered orally on day 7. Body weight, fecal redox potential and pH were measured on days 1, 3, 5, 7, 10, 12, and 14. *C. rodentium* load was quantified via serial dilution and plate counting on MacConkey agar on days 7, 10, 12, and 14.

The anti-colonization mechanisms and functional efficacy of coated versus uncoated antioxidants were further compared in Experiment 2. SD rats were randomly assigned to four groups (n = 6): Con, Con-Cr, FA-Cr, and FAC-Cr. After one week of acclimation, rats in FA-Cr and FAC-Cr groups received respective antioxidant-supplemented diets, while Con and Con-Cr were fed the basal diet. *C. rodentium* was administered orally on day 7. Body weight, fecal redox potential, and pH were monitored throughout the experiment. On day 14, rats were sacrificed via CO_2_ asphyxiation. Colonic chmye and mucosa were collected for *C. rodentium* enumeration and downstream metagenomic analysis.

### 2.5 Measurement of redox potential and pH

Fresh fecal and intestinal content samples were collected in CO_2_-flushed bags, with sampling completed within 30 min. Samples were immediately sealed, homogenized, and redox potential was measured as described previously [15]. Briefly, fresh fecal samples were immediately collected for redox potential measurement using an ST300/B oxidation-reduction potential electrode. For fecal pH measurements, fresh fecal samples were collected and pH was determined using a hand-held pH meter (Hanna, Italy).

### 2.6 Determination of FA content based on HPLC analysis

Approximately 0.1 g of colonic contents was accurately weighed and homogenized in a mortar. Subsequently, 1 mL of pre-cooled Reagent I was added, and the mixture was transferred to an Eppendorf tube. Ultrasonic extraction was performed for 60 min. The sample was then centrifuged at 10 000×*g* for 10 min, and the resulting supernatant was collected. The residue was re-extracted with 0.5 mL of Reagent I by ultrasonication for an additional 20 min, followed by centrifugation under the same conditions. Both supernatants were combined and diluted to a final volume of 1.5 mL with Reagent I. The resulting solution was filtered through a 0.22 μm syringe filter prior to HPLC analysis. Chromatographic separation was carried out using a Rigol L3000 HPLC system equipped with a Compass-C18 reversed-phase column (250 mm × 4.6 mm, 5 μm). The mobile phase consisted of methanol: acetonitrile: acetic acid: water (15:15:5:65, v/v). The flow rate was set at 0.8 mL/min, and the column temperature was maintained at 25 °C. Detection was performed at 280 nm with an injection volume of 10 μL. The total run time was 20 min.

### 2.7 Determination of serum inflammatory factors

After serum collection, the concentrations of inflammatory factors, including Crp, TNF-α, IL-1β and IL-6 were quantified using commercial enzyme-linked immunosorbent assay (ELISA) kits. The ELISA kits were procured from Nanjing Aoqing Biology and the method was employed to generate colorimetric signals by capturing the interaction between target antibodies and antigens for quantitative analysis. Specifically, the assays were performed strictly according to the manufacturer's instructions, with each sample measured in triplicate to ensure data reliability.

### 2.8 Real time PCR quantitative analysis of *C. rodentium* load

*C. rodentium* load was quantified by real-time PCR using DNA extracted from colonic digesta. A standard curve (0–2.1 × 10^10^ CFU) was established using *C. rodentium* culture DNA extracted with the DNA Kit (Qiagen). Target-specific primers for the *EspB* gene (*EspB*-F: 5′-ATGCCGCAGATGAGACAGTTG-3’; *EspB*-R: 5′-GTCAGCAGCCTTTTCAGCTA-3′) were employed as previously validated [24]. Reaction mixtures contained 12.5 ng colonic DNA or standard DNA, 10 mM primers, and SYBR select master mix (Roche). Thermal cycling protocol: Initial denaturation at 95 °C for 10 min, followed by 40 cycles of 95 °C (15 s), 60 °C (60 s), and 72 °C (30 s) using a ViiA 7 Real-Time PCR System (Applied Biosystems). CFU values were extrapolated from CT-based standard curves and normalized to CFU/g digesta using sample mass recorded during DNA extraction.

### 2.9 Determination of mucus barrier function

#### 2.9.1 Mucus layer thickness measurement

Colonic segments were excised and meticulously cleared of mesenteric fat. After luminal ligation with surgical sutures, tissues were fixed in Carnoy's solution (≤12 h) [25], dehydrated through graded ethanol series, and paraffin-embedded. For mucus layer characterization, 5 μm sections were dual-stained with Alcian Blue (AB; Solarbio, G1285) following manufacturer protocols. Mucosal thickness was quantified using Adobe Photoshop 2021 (v22.0.0) with calibrated measurement tools [26].

#### 2.9.2 Immunofluorescence observation

Immunofluorescence was performed on consecutive sections (4 μm) after deparaffinization and antigen retrieval. Sections were blocked (1 h, RT) and incubated overnight (4 °C) with *anti*-Muc2 primary antibody (Servicebio, GB11344). Alexa Fluor-conjugated secondary antibodies and DAPI counterstain (Beyotime, A-11008) were applied for 1 h and 10 min respectively [27]. Fluorescence intensity was quantified via Image J (v1.53) using standardized threshold parameters.

#### 2.9.3 Expression of genes related to mucus secretion

To assess mucus secretion-related gene expression, total RNA was extracted from colon tissues using a dedicated kit (Qiagen, Germantown, MD, USA) [28]. RNA was reverse-transcribed into cDNA using a reverse transcription kit (Abm, G592, Zhenjiang, China). mRNA expression levels of *Muc2, Muc5ac, Tff1,* and *Tff3* were quantified on a real-time PCR system using primers listed in Table S3. Relative expression was calculated by the 2^ΔΔCt^ method and normalized to the expression of GAPDH.

### 2.10 DNA extraction, 16S rRNA gene and metagenomic sequencing

Fecal microbial DNA was extracted using the E.Z.N.A.® Fecal DNA Kit (Omega Bio-tek), followed by amplification of the 16S rRNA V3–V4 regions with primers 341F/806R and paired-end sequencing (2 × 250 bp) on the Illumina MiSeq platform. Operational taxonomic units (ASVs) were delineated at 97 % identity threshold using USEARCH v10.0. Microbial diversity was assessed through Shannon index and richness and Bray-Curtis-based PCoA. Cohort-specific community overlaps were visualized via Venn diagrams and UpSet plots [29]. For metagenomic profiling, 150 bp paired-end libraries were sequenced on HiSeq X Ten (Illumina) [30]. Raw reads underwent quality control (Trimmomatic v0.39), reference alignment (BWA v0.7.17), and de novo assembly (MEGAHIT v1.2.9; >500 bp contigs). Open reading frames were predicted with Prodigal v2.6.3, dereplicated (CD-HIT v4.8.1), and taxonomically classified via DIAMOND v2.0.15 against NCBI-NR [15]. Functional annotation employed KofamScan for KEGG orthology mapping, with expression quantified as transcripts per million (TPM).

### 2.11 Bacterial co-culture

To further validate the inhibition interaction between beneficial and pathogenic bacteria observed *in vivo*, a co-culture experiment was performed using *L. reuteri* and *C*. *rodentium in vitro*. The *L. reuteri* strain used for the challenge experiment was obtained from our laboratory. *L. reuteri* and *C. rodentium* were used to evaluate competitive interactions under controlled redox conditions [31]. Overnight cultures of each strain were grown anaerobically in basal solution [32] broths at 37 °C. Based on the principle of the Winogradsky column [33], where natural oxygen diffusion and microbial metabolism create redox potential gradients, this liquid shake-flask culture system was designed to simulate the behavior of gut microbes under different redox environments. Different redox potentials were established by using Na_2_S·9H_2_O in combination with the redox indicator resazurin. For co-culture experiments, bacterial suspensions were adjusted to an initial OD_600_ of 0.1 and mixed in equal volumes in culture media with different redox potentials. The redox potential of the medium was modulated using redox electrode to simulate low and high redox environments. At designated time points, samples were collected for bacterial quantification by CFU counting, and for transcriptional analysis of *C. rodentium tir* and *ler* genes via RT-qPCR (Table S3). Data were normalized to *rpoA* for bacterial virulence factor detection, and analyzed using the 2^–ΔΔCT^ method [31]. Competitive effects were determined by comparing *C. rodentium* growth and virulence gene expression in the presence versus absence of *L. reuteri* under different redox conditions.

### 2.12 Statistical analysis

Longitudinal effects were analyzed using a two-way repeated measures ANOVA implemented in SPSS 26.0, with time as the within-subjects factor and group as the between-subjects factor, including the time × group interaction term. Intergroup differences were evaluated via one-way ANOVA with Tukey post hoc tests. Differences in microbial genera/pathways were assessed using Wilcoxon rank-sum tests. Data are expressed as mean ± SD (*P* < 0.05 considered significant). Spearman's rank correlation analysis was performed using the R package psych. Detailed statistical methods are provided in figure legends.

## 3 Results

### 3.1 Targeted regulation of intestinal redox potential by coated EQ and FA

Colon targeted delivery systems for EQ and FA were developed using fluidized bed encapsulation technology (Fig. 1A). Post-processing characterization revealed uniform nanoparticle formation (252.3 ± 18.7 nm; Fig. 1B). Under gastric conditions (pH 2), FAC exhibited a near-neutral zeta potential (−1.2 mV) in contrast to EQC (−24.8 mV; Fig. 1C; *P* < 0.05). This physicochemical property facilitated the preferential colonic release of FAC through pH-responsive dissociation. *In vitro* antioxidant assessments revealed differential activity retention, with EQC maintaining 89.4 % of the DPPH radical scavenging capacity of native EQ, whereas FAC showed 62.3 % reduction compared to unmodified FA (Fig. 1D; *P* < 0.05). Both coated antioxidants exhibited concentration-dependent FRAP activity (Fig. 1E; *P* < 0.05). Subsequent *in vivo* animal trials (Fig. 1F; *P* < 0.05) demonstrated that both low- and high-concentration FAC effectively reduced intestinal redox potential throughout the intervention period (Fig. 1G; *P* < 0.05), whereas EQC exhibited a gradual reduction effect on the redox potential from day 3 to day 7 (Fig. 1H; *P* < 0.05). Compared to uncoated antioxidants, both coated antioxidants showed a significantly enhanced redox potential-lowering capacity, confirming that colon-targeted antioxidant delivery effectively regulated the intestinal redox potential through controlled release mechanisms.

### 3.2 Maintenance of a low intestinal redox potential and reduction of *C. rodentium* load by coated EQ and FA

**Fig. 2:**
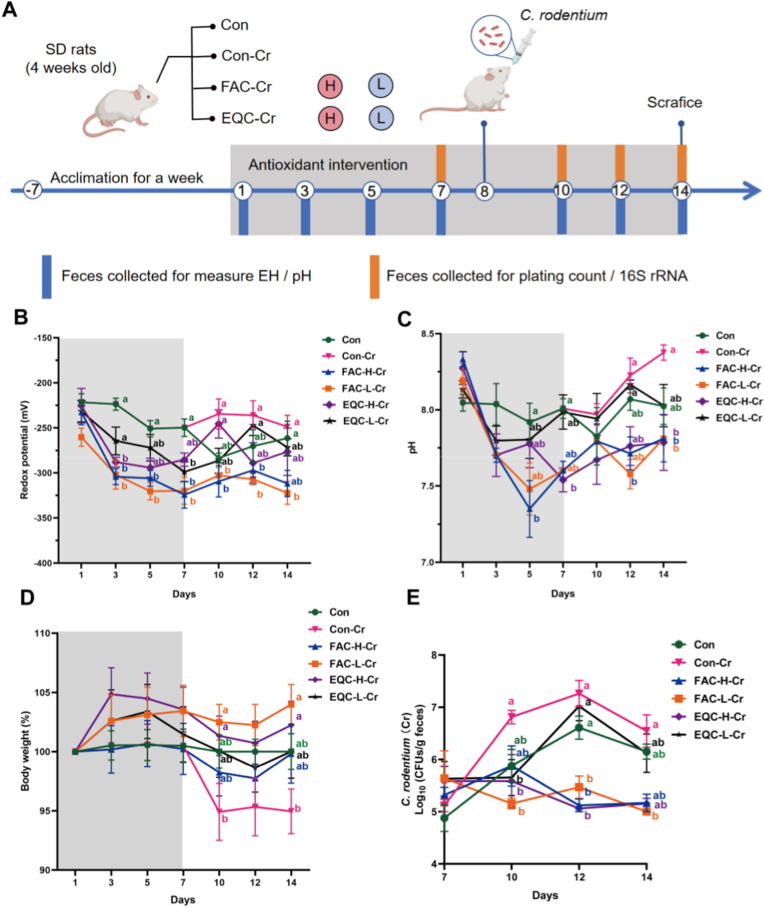
Coated EQ and FA maintained a low intestinal redox potential and reduced *C. rodentium* load. (A) Experimental design and group arrangement. n = 6 in Con and Con-Cr groups. n = 5 in EQC-Cr and FAC-Cr groups. (B, C, D, E) Dynamic changes in redox potential, pH values, body changes and *C. rodentium* plate count.

Subsequently, the ability of coated chemical antioxidants and natural antioxidants to regulate intestinal redox potential and enhance resistance against pathogens was compared (Fig. 2A). Repeated measures ANOVA revealed a significant main effect of time on redox potential, with the potential values decreasing over time in all groups (Con: −1.82/day). Among them, the FAC-H-Cr group had the fastest overall decline rate of −5.94/day, which was significantly faster than that of the other groups (Fig. 2B; *P* < 0.05). The effect of coated antioxidants on intestinal redox potential was evaluated, the results of which showed that the intestinal redox potential was continuously reduced before pathogen challenge in FAC groups, with slightly better efficacy observed in the low-concentration group, indicating that the redox potential-lowering effect of FAC was dose-independent (Fig. 2B; *P* < 0.05). After *C. rodentium* challenge, FAC-Cr (H/L) exhibited significant regulatory effects on intestinal redox potential. Concurrently, consistent fluctuations in intestinal pH were observed (Fig. 2C). On days 10 and 14, the body weight change rate was significantly increased in the FAC-L-Cr group compared with the Con-Cr group, indicating that FAC-L-Cr effectively reversed the *C. rodentium*-induced weight loss (Fig. 2D; *P* < 0.05). Continuous observation of post-challenge fecal *C. rodentium* load revealed that on day 10, the fecal *C. rodentium* load was significantly reduced in both FAC-L-Cr and EQ-L-Cr groups compared with the Con-Cr group (Fig. 2E; *P* < 0.05). On days 12, the *C. rodentium* load was significantly decreased in the FAC-H-Cr, FAC-L-Cr, and EQ-H-Cr groups (Fig. 2E; *P* < 0.05). These results provided evidence that coated antioxidants effectively reduced intestinal redox potential and counteract the growth performance impairment caused by *C. rodentium* infection.

### 3.3 Consistent alteration of intestinal microbiol structure by coated EQ and FA

**Fig. 3:**
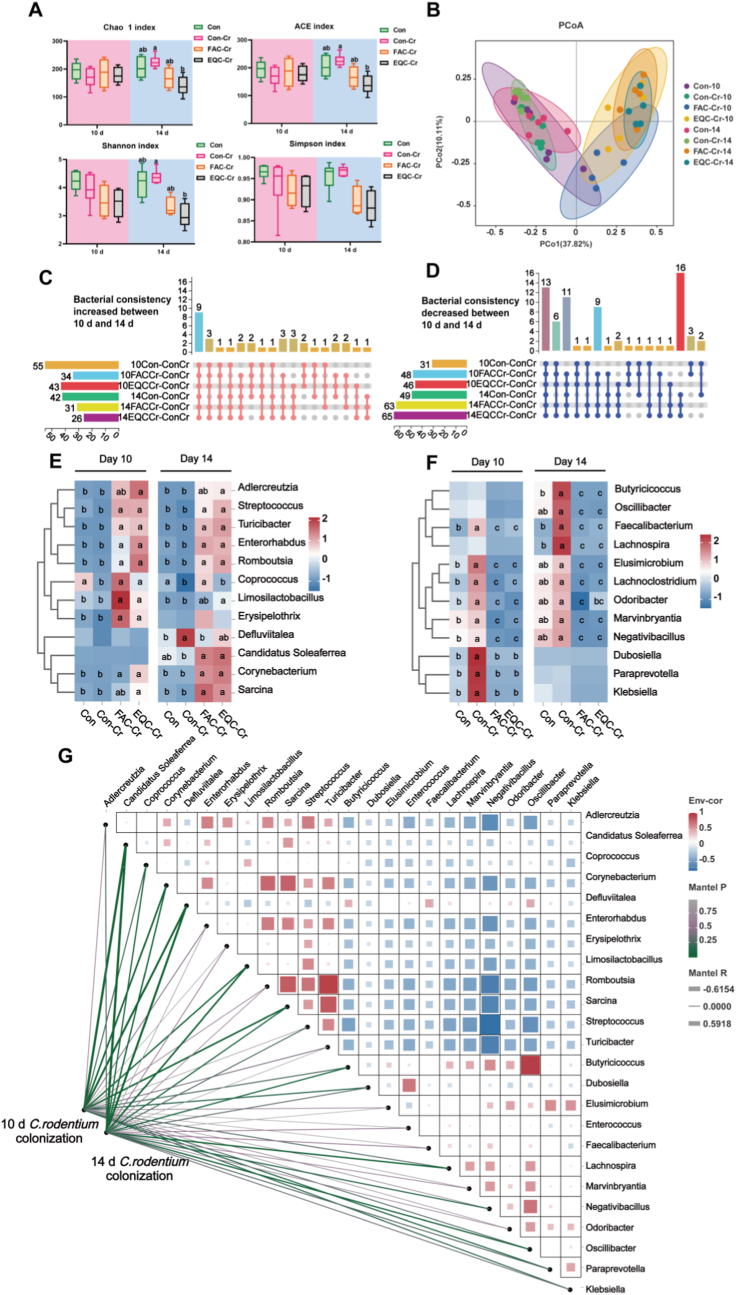
Coated EQ and FA consistently altered the structure of intestinal microbiota. n = 6 in Con and Con-Cr groups. n = 5 in EQC-Cr and FAC-Cr group. (A, B) The α diversity and PCoA analysis of the intestinal microbiota on the 10 and 14 days. (C, D, E, F) UpSet diagrams based on bacterial genera that exhibited consistent increased and decreased across different groups compared to the Con-Cr group. (G) Mantel test revealed the compositional changes of consistency-increasing and consistency-decreasing bacteria and *C. rodentium* load. The width of the lines indicates the mantel's r statistic value of the corresponding distance correlation, and the color of the lines represents the statistical significance. The gradient color bar represents the spearman correlation coefficient of pairwise comparisons between *C. rodentium* load and different bacterial genera.

During the 10-day treatment period, FAC-Cr and EQC-Cr had no significant effect on the α-diversity of fecal microbiota. However, on day 14, EQC-Cr significantly reduced Chao1, ACE and Shannon indexes of fecal microbiota (Fig. 3A; *P* < 0.05), while the microbiota structure of FAC-Cr became closer to that of the Con group. Meanwhile, the microbiota compositions of FAC-Cr and EQC-Cr were markedly distinct from those of Con and Con-Cr groups (Fig. 3B). Using UPset venn diagrams to capture microbiota information across treatment groups, the intersections of microbiota under different treatments were detected. On both days 10 and 14, nine genera were consistently increased across treatment groups compared to the Con-Cr group (Fig. 3C), while 13 genera were consistently decreased (Fig. 3D). The FAC-Cr and EQC-Cr groups showed a significant increase in *Streptococcus, Turicibacter, Enterorhabdus,* and *Romboutsia* abundances at both time points. On day 10, *Limosilactobacillus* abundance significantly increased in the FAC-Cr and EQC-Cr groups, while on day 14, *Limosilactobacillus* abundance rose notably in the EQC-Cr group (Fig. 3E; *P<0.05*). Notably, the FAC-Cr and EQC-Cr groups exhibited significant increases in *Faecalibacterium, Elusimicrobium, Lachnoclostridium,* and *Negativibacillus* abundances on both days 10 and 14 (Fig. 3F; *P<0.05*). On day 14, the abundances of *Butyricicoccus* and *Lachnospira* in the FAC-Cr and EQC-Cr groups were significantly higher than those in the Con-Cr group, though no significant difference was observed on day 10 (Fig. 3F; *P>0.05*). The mantel test was subsequently employed to correlate the consistently increased bacteria with the consistently decreased bacteria and to analyze their association with the *C. rodentium* load matrix (Fig. 3G). Correlation analysis demonstrated a negative association between consistently increased bacteria and consistently decreased bacteria, suggesting that there might be competitive interactions among these microbiota. To be specific, the abundances of *Streptococcus, Romboutsia, Limosilactobacillus, Sarcina*, and *Turicibacter* were significantly negatively correlated with consistently decreased bacterial taxa in the gut (Fig. 3G; *P<0.05*). Mantel test further indicated that *Limosilactobacillus, Romboutsia* and *Sarcina* were significantly negatively correlated with *C. rodentium* load at both 10-day and 14-day treatment timepoints (Fig. 3G; *P<0.05*). These findings suggested that the coating antioxidants altered the gut microbiota structure, consistently enhancing the abundance of beneficial bacteria. Notably, *Limosilactobacillus*, *Romboutsia* and *Sarcina* may compete with *C. rodentium* microbiota, potentially through niche competition or metabolic interactions.

### 3.4 Effective weakening of the colonization of *C. rodentium* and the inflammatory response by coated FA

**Fig. 4:**
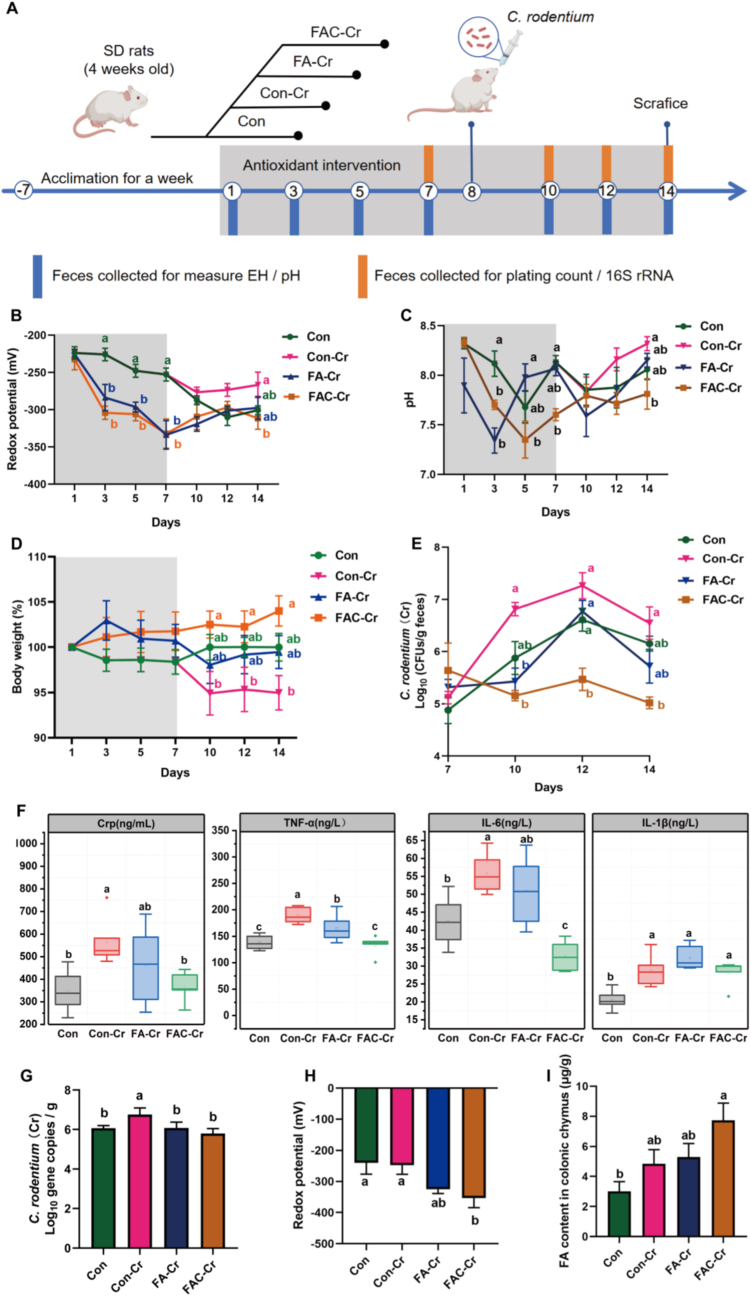
Coated FA weakened colonization of *C. rodentium* and the inflammatory response. (A) Main experimental design. n = 6 in Con, Con-Cr and FA-Cr groups. n = 5 in FAC-Cr group. (B, C, D, E, F) Antioxidants improved the redox potential and pH, mitigated weight loss, reduced *C. rodentium* colonization and alleviated inflammation. (G, H, I) The *C. rodentium* load, redox potential and FA content in colonic chyme.

The inhibition of *C. rodentium* colonization efficacy of coated and uncoated antioxidants were further compared in Fig. 4A. Both interventions led to a significant decline in redox potential (Fig. 4B). FAC-Cr and FA-Cr also caused fluctuations in intestinal pH (Fig. 4C). Compared with FA-Cr, FAC-Cr significantly reversed pathogen-induced weight loss. On days 10, 12, and 14 of the intervention period, the weight change rate in the FAC-Cr group was significantly higher than that in the Con-Cr group, with the most pronounced reversal of weight loss observed on day 14 (Fig. 4D; *P<0.05*). In contrast, FA-Cr failed to mitigate weight loss. Concurrently, FAC-Cr markedly reduced intestinal *C. rodentium* load. During the period from day 10 to day 14, the *C. rodentium* load in the FAC-Cr group showed no significant fluctuations over time and remained consistently lower than that in the Con-Cr group, aligning with redox potential variations (Fig. 4E; *P<*0.05). Post-sacrifice analysis revealed that FAC-Cr significantly lowered serum Crp level. Both FAC-Cr and FA-Cr reduced TNF-α level, while FAC-Cr specifically decreased serum IL-6 levels. However, neither treatments significantly altered IL-1β level compared with Con-Cr (Fig. 4F; *P<0.05*). Absolute quantification of *C. rodentium* in colonic chyme confirmed that FAC-Cr significantly reduced *C. rodentium* load compared with Con-Cr (Fig. 4G; *P* < 0.05). Further measurements of colonic redox potential post sacrifice showed that FAC-Cr reduced redox potential more effectively than FA-Cr (Fig. 4H; *P* < 0.05). Meanwhile, the FA content in the FAC-Cr group was significantly increased compared to the other groups (Fig. 4I; *P* < 0.05), indicating that FAC-Cr had an excellent targeted release effect in the colon.

### 3.5 Specific preservation of the integrity of mucosal barrier by coated FA

**Fig. 5:**
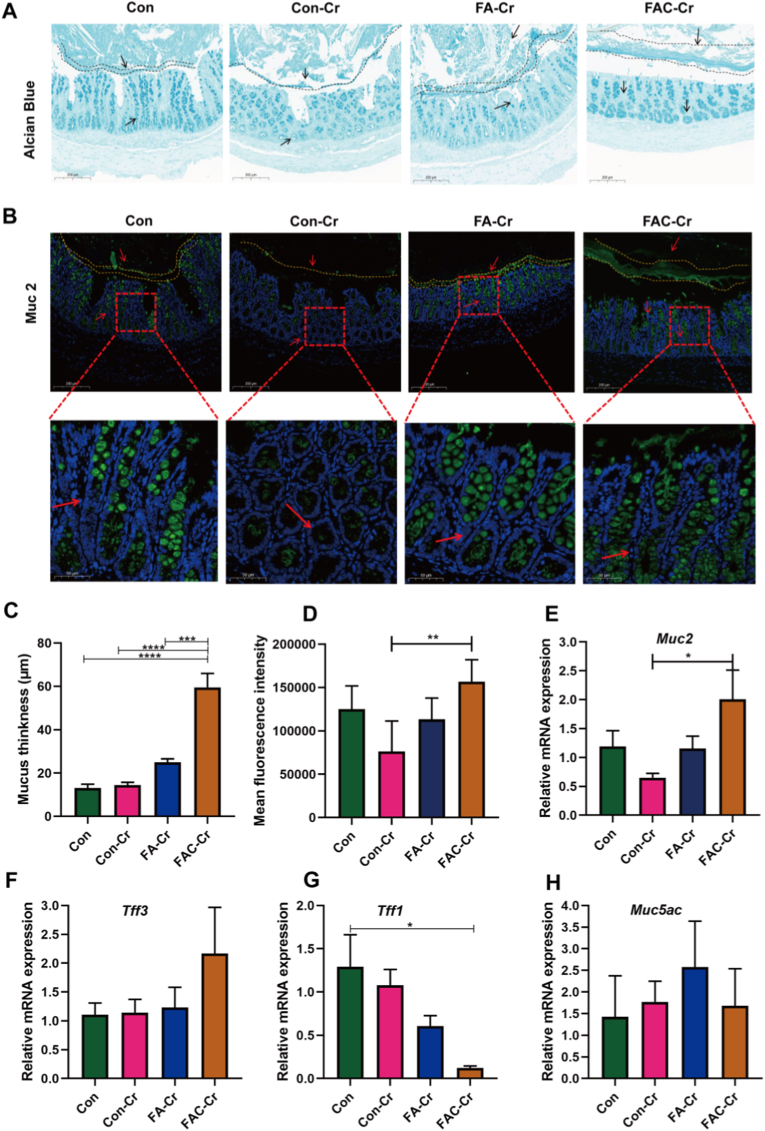
Coated FA preserved mucosal barrier integrity. (A) AB staining images of the distal colons of mice. n = 6 in Con, Con-Cr and FA-Cr groups. n = 5 in FAC-Cr group. Scale bars: 100 μm. (B) Immunofluorescence staining of mouse colon sections with *anti*-Muc2. Scale bars: 100 μm. The red box denoted the selected region of interest that had been magnified and the dashed boxes highlight. (C) Mucus layer thickness measurement. (D, E, F, G, H) Immunofluorescence results of mucus secretion and relative mRNA expression of *Muc2 Tff3 Tff1 Muc5ac* in the rat colon. *P < 0.05, **P < 0.01, ***P < 0.001, ****P < 0.0001.

Subsequently, the effect of antioxidant supplementation on the mucus production in rats was investigated. As shown in Fig. 5A, the colonic mucus layer of rats in the FAC-Cr group remained intact with markedly increased thickness, while that in the Con-Cr group was loosely structured with reduced thickness, suggesting that FAC-Cr significantly affected the homeostasis of intestinal mucus layer. Immunofluorescence staining of colonic tissues revealed that the *Muc2* fluorescence signal in the FAC-Cr group was significantly stronger than that in the Con-Cr group (Fig. 5B). FA-Cr also showed partial protective effects on mucus layer thickness and *Muc2* integrity, but its fluorescence signal intensity remained weaker than that of FAC-Cr (Fig. 5C). FAC-Cr significantly increased the length of intestinal fluorescence signal (Fig. 5D; *P* < 0.05). Further analysis of key mucus-related key genes revealed that FAC-Cr significantly upregulated the expression of intestinal *Muc2* gene (Fig. 5E; *P* < 0.05), while downregulated the gene expression of *Tff1* (Fig. 5G; *P* < 0.05). No significant changes were observed in the expression genes of *Tff3* and *Muc5ac* (Fig. 5F–H; *P* > 0.05). The differential regulation of mucin genes (*Muc2* and *Muc5ac*) and trefoil factors (*Tff1* and *Tff3*) indicated a selective mechanism by FAC in mucus homeostasis maintenance.

### 3.6 Increased abundance of beneficial bacteria and reduced abundance of Enterobacteriaceae in different ecological niches by coated FA

**Fig. 6:**
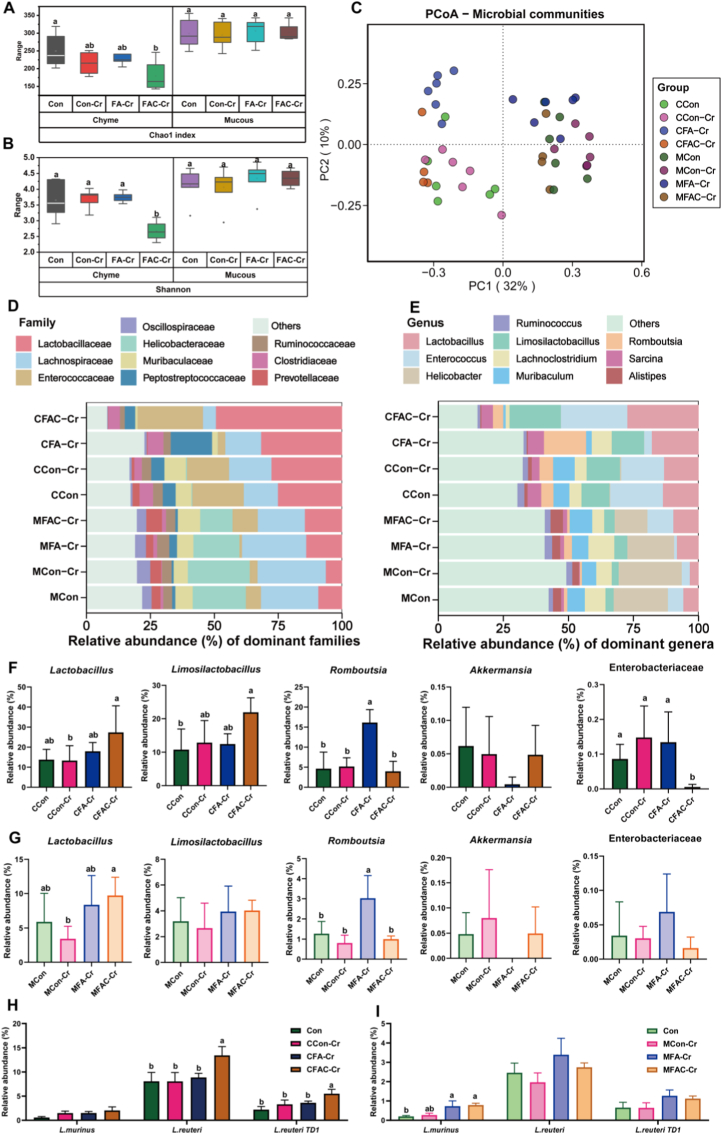
Coated FA increased the abundance of beneficial bacteria and inhibited Enterobacteriaceae in different ecological niches. n = 6 in Con, Con-Cr and FA-Cr groups. n = 5 in FAC-Cr group. (A, B) The box plot showed the α diversity of chyme and mucous samples in different groups. (C) The PCoA analysis indicated significant separation of the microbiota structure among groups. (D, E) Identification of microbiota composition differences and marker microbiota. (F, G, H, I) Composition diagram of differential microbiota in colonic chyme and mucous.

The effects of FA and coated FA on the colonic microbiota were further compared. In the mucosal samples, there were no significant changes observed in either the Chao1 or Shannon diversity indexes. In the chyme samples, the FAC-Cr group exhibited a significantly lower Chao1 index compared to the Con group, while the Shannon index in the chyme of the FAC-Cr group was significantly lower than that of other groups (Fig. 6A and B; *P* < 0.05). PCoA analysis demonstrated a distinct separation between chyme and mucosa along the PC1 axis (explaining 32 % of the variation). The distribution along the PC2 axis (accounting for 10 % of the variation) partially captured inter-group differences (Fig. 6C). The stacked bar chart showed differences in relative abundance at the family and genus levels. Lactobacillaceae was more abundant in the FAC-Cr group (both chyme and mucosa), particularly in chyme. Lachnospiraceae and Ruminococcaceae were less abundant in the Con-Cr group, especially in mucosa (Fig. 6D). In chyme, *Lactobacillus* abundance was significantly higher in the FAC-Cr group than in the Con and Con-Cr groups. In the FAC-Cr group, *Limosilactobacillus* abundance was higher in the mucosa but less pronounced than in the chyme (Fig. 6E). *Lactobacillus* abundance in the chyme of the FAC-Cr group sharply increased compared to the Con and Con-Cr groups (Fig. 6F; *P* < 0.05). In comparison with other groups, *Limosilactobacillus* abundance in the chyme was significantly increased in the FAC-Cr group (Fig. 6F; *P* < 0.05). However, such changes were not significant in the mucosa. Notably, Enterobacteriaceae was inhibited in both chyme and mucosa in the FAC-Cr group, with the inhibitory effect being more pronounced in the chyme (Fig. 6G; *P* < 0.05). Consistent results were observed at the species level, where *L. reuteri* and *L. reuteri TD1* abundance was significantly enriched in both FAC-Cr groups compared with the other groups (Fig. 6H; *P* < 0.05). *L. murinus* abundance was reduced in the Con group (Fig. 6I; *P* < 0.05), whereas *L. reuteri TD1* abundance showed a moderate but nonsignificant increase in the MFAC-Cr group. These results suggested that FAC may enhance colonization resistance by increasing the competition between beneficial bacteria and pathogenic bacteria.

### 3.7 Coated FA inhibited Enterobacteriacea colonization by altering the function of intestinal microbiota

**Fig. 7:**
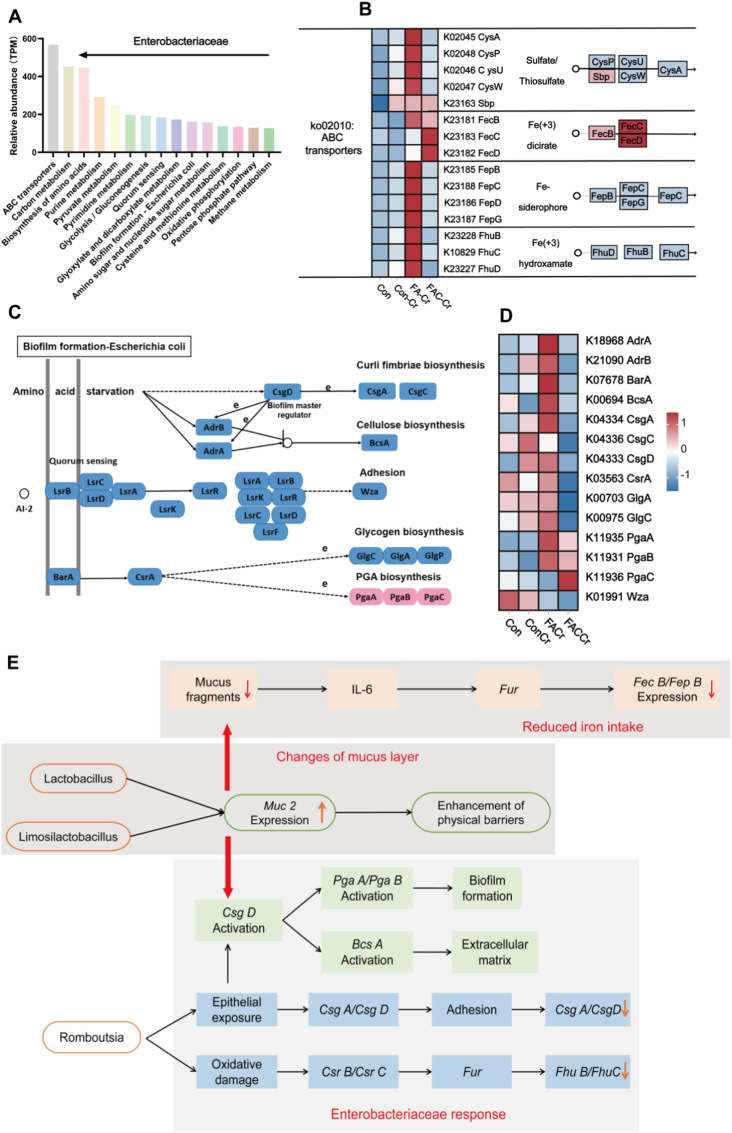
Coated FA affected bacterial colonization by altering the function of intestinal microbiota. n = 4 per group. (A) The relative abundance of transport proteins related to different metabolic pathways in Enterobactericeae*.* (B) List of ABC transporter-related genes in the ko02010 pathway. (C) Schematic diagram of the regulatory pathways related to biofilm formation. (D) Heatmap of KO genes associated with biofilm formation in the colonic metagenome. (E) The functional association between *Akkermansia*, *Lactobacillus*, *Romboutsia*, and the functional analysis.

The pathways enriched in Enterobacteriaceae were ranked through gene enrichment analysis (Fig. 7A). KEGG Orthology analysis revealed that functional genes were involved in ABC transporters, SCFA-related pathways (Pyruvate metabolism, Carbon metabolism, Quorum Sensing, and Biofilm Formation), Amino sugar and nucleotide sugar metabolism, and Oxidative phosphorylation. Enterobacteriaceae enhanced their nutrient acquisition capability by highly expressing ABC transporters. However, FAC-Cr inhibited sulfur metabolism by blocking the gene cluster (*CysA/CysP/CysU*) responsible for sulfate/sulfite uptake and disrupted osmotic pressure homeostasis by interfering with multi-ion transmembrane transport channels. Conversely, FAC-Cr promoted efficient capture of free iron ions via upregulation of the siderophore system (*FecB/FepB/FhuD*) (Fig. 7B). FAC-Cr remodeled the biofilm formation pathway in *Escherichia coli*, which was regulated by AI-2 quorum sensing and the *CsgD*/c-di-GMP signaling hub. In the FA-Cr group, key genes (*AdrA/BcsA/CsgA*) were highly expressed, promoting cellulose synthesis (*BcsA*), curli fimbriae assembly (*CsgA*), and polysaccharide adhesin secretion (*PgaA/PgaB/PgaC/PgaD*). By contrast, in the FAC-Cr group, these genes were significantly downregulated, thereby weakening the structural stability of biofilm (Fig. 7C). Additionally, FAC-Cr suppressed glycogen biosynthesis genes (*GlgA/GlgC*) and capsule export protein (*Wza*), thereby effectively blocking the formation of extracellular polymeric substances (Fig. 7D). The functional associations between *Akkermansia, Lactobacillus, Romboutsia, Limosilactobacillus* and the host intestinal mucosa were analyzed. Probiotics (*Lactobacillus/Limosilactobacillus/Romboutsia*) promoted *Muc2* expression and secretion through glycolysis/pyruvate metabolism (Fig. 7E). Enterobacteriaceae triggered biofilm formation using quorum sensing and enhanced colonization through glycerophospholipid metabolism and oxidative phosphorylation, thereby creating a negative feedback loop for the host barrier.

### 3.8 Lower redox potential further drived the competition between *L. reuteri* and *C. rodentium*, thereby reducing *C. rodentium* virulence

**Fig. 8:**
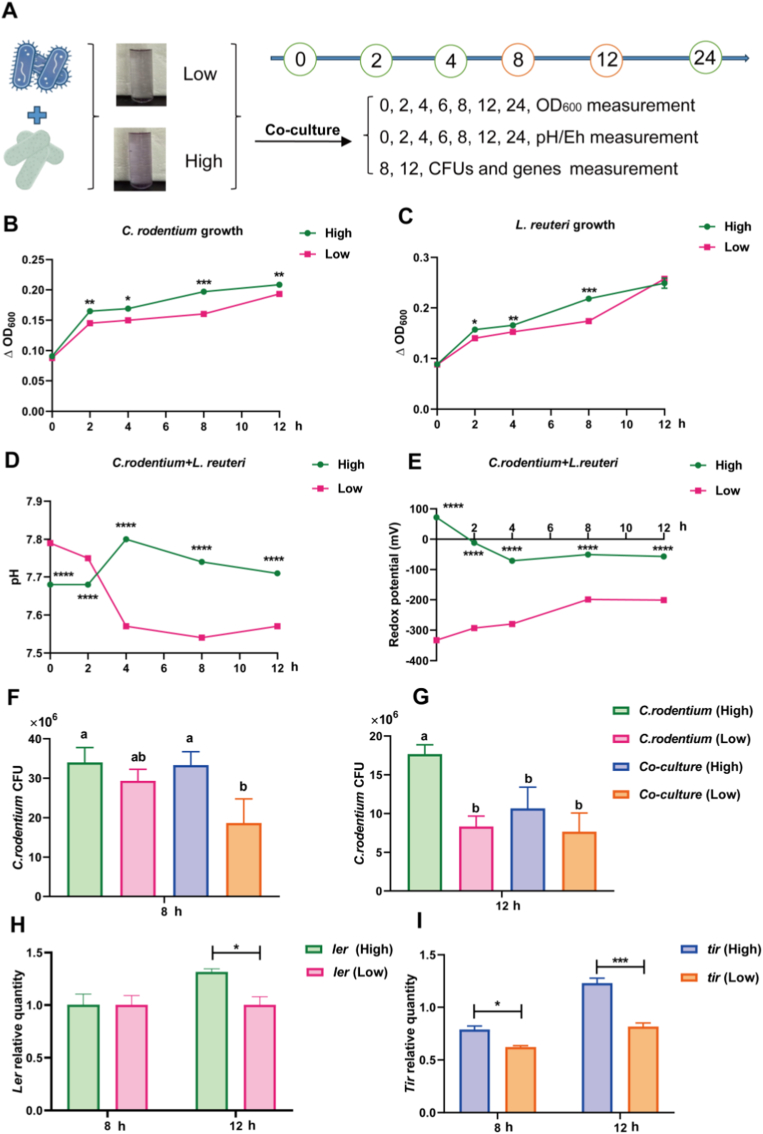
*L. reuteri suppressed C. rodentium growth and virulence under lower redox potential.* n = 3 per group. (A) An *in vitro* fermentation system was established to simulate different redox potentials based on the principle of the Winogradsky column. (B, C) *C. rodentium* and *L. reuteri* were cultured alone in the fermentation broth. (D, E) Changes in pH and redox potential were monitored in the co-culture system. (F, G) The bacterial load of *C. rodentium* was determined in co-culture systems with different redox potentials at 8 h and 12 h. (H, I)The relative expression of the virulence genes *tir* and *ler* in *C. rodentium* was assessed in co-culture systems with different redox potentials at 8 h and 12 h *P < 0.05, **P < 0.01, ***P < 0.001, ****P < 0.0001.

*In vitro* co-culture systems adjusted to high or low redox potential, distinct growth and virulence phenotypes were observed (Fig. 8A). Both bacterial strains were able to grow separately in the culture systems (Fig. 8B and C). In the co-culture system, *L. reuteri* significantly inhibited the growth of *C. rodentium* compared with its monoculture, and this inhibitory effect was further enhanced under the lower redox condition (Fig. 8D and E; *P* < 0.05). Correspondingly, bacterial counts in the low redox potential co-culture showed a pronounced suppression of *C. rodentium* CFUs (Fig. 8F and G; *P* < 0.05), suggesting that the reducing conditions conferred a competitive advantage to *L. reuteri*. Furthermore, the lower redox potential environment significantly downregulated the expression of *C. rodentium* virulence genes (*ler* and *tir*) compared with the high redox potential (Fig. 8H and I; *P* < 0.05), implying that the pathogenic virulence potential was attenuated. These findings were consistent with the results in the animal experiment, further confirmed that a lower redox potential environment allowed *L*. *reuteri* to gain a competitive advantage over *C*. *rodentium* and suppressed the virulence genes expression of *C*. *rodentium*. These results highlighted the pivotal role of redox regulation in shaping microbial competition and controlling pathogen virulence within the gut ecosystem.

## 4 Discussion

In this study, a novel strategy, involving the regulation of intestinal redox potential through controlled release of coated antioxidants to limit pathogenic bacteria colonization, was systematically proposed for the first time and experimentally validated. The results demonstrated that coated FA with a protective layer significantly reduced the intestinal redox potential levels, thereby disrupting the ecological niche adaptation capacity of Enterobacteriaceae. Potential mechanisms include the inhibition of biofilm formation and the suppression of functional expression of the ABC transport system in Enterobacteriaceae, which collectively constrain their ability to colonize and proliferate. The intestinal redox potential reflects the electron transfer capacity within the gut and plays a pivotal role in shaping gut microbial composition and protecting host health [34]. A pronounced redox potential gradient exists in the gastrointestinal tract: aerobes and facultative anaerobes predominate in highly oxidative (high-redox potential) niches, whereas obligate anaerobes reside in reductive (low-redox potential) regions. Consequently, maintaining colonic redox balance is critical for microbiota-host immune interactions and disease susceptibility [35]. Dietary antioxidants could attenuate oxidative stress by shifting redox potential toward a more reduced state, thereby promoting gut health [3]. Their broader adoption, however, is constrained by complex extraction processes, supply limitations, higher costs, and degradation during feed processing. Fortunately, coated techniques address these constraints by protecting bioactivity and enabling controlled release [36]. Here, controlled - release antioxidants using formulations of similar particle size under simulated gastrointestinal pH were compared. Compared with EQC, FAC was more effective in lowering colonic redox potential. These results indicate that the controlled release of FA more effectively modulates intestinal redox potential, and animal experimentation further confirmed that the activity of the coated antioxidant protected it from premature degradation or deactivation, which was correlated with a reduced *C. rodentium* load and the mitigation of infection - induced weight loss.

Given the crucial role of gut microbiota in modulating host health and pathogen resistance, the mechanisms by which coated EQ and FA affect the gut microbial community under the low-redox potential environment established by controlled release were further investigated. Under *C. rodentium* challenge, the microbiota of coated FA closely resembled uninfected control in overall diversity, whereas coated EQ showed greater dysbiosis. Both coated EQ and FA increased the abundance of *Turicibacter, Enterorhabdus, Romboutsia, Limosilactobacillus*, which were typically linked to health-promoting activities [3,4]. However, coated FA had downregulated genera *Faecalibacterium, Lachnospira, Elusimicrobium, Lachnoclostridium,* and *Klebsiella*, all of which are associated with branched-chain amino acid metabolism, SCFAs production, biofilm formation, and inflammatory responses [37]. Interestingly, coated FA treatment enriched *Limosilactobacillus, Romboutsia*, and *Sarcina*, which were found to have a negative correlation with *C. rodentium* load. The enriched *Lactobacillus, Limosilactobacillus, Romboutsia,* and *Sarcina* suppress Enterobacteriaceae colonization via ecological competition mechanisms, including nutrient and adhesion site competition, organic acid production, and enhancement of mucosal barrier function [38,39]. These mechanisms have been well documented as critical components of microbiota-mediated colonization resistance against enteric pathogens.

Notably, our findings suggest that coated FA had a better effect in reducing the *C. rodentium* load than FA. The initial step of pathogenic bacteria adhesion in the gut involves contact with the intestinal mucus layer. Given the pivotal role of the mucus barrier in host defense, it was next explored whether the modulation of redox potential by coated FA affects mucin expression and mucus integrity, thereby contributing to the reduced pathogen adhesion and colonization. The mucus layer is synthesized and secreted by goblet cells, and an increase in its thickness may indicate a strengthened barrier, which correlates with reduced microbial penetration and improved epithelial protection [40]. In the colonic environment, the mucus layer is structured into two distinct zones: an inner layer that is impermeable to bacteria and an outer layer that supports microbial colonization. An increased overall thickness of the mucus layer may enhance the protective capacity of the inner layer and limit pathogen exposure [41]. Disruption of the mucus layer is a critical indicator of heightened susceptibility to pathogenic bacteria invasion. *C. rodentium* possesses mucin-degrading capability and establishes an infection by disrupting the mucus layer. Barrier disruption initially facilitates *C. rodentium* colonization, which in turn accelerates mucin degradation and perpetuates a cycle of epithelial damage and pathogen persistence [42]. These findings further support the hypothesis that coated FA–induced thickening of the mucus layer may contribute to improved intestinal barrier function through a comparable mechanism. *Muc2* gene is the predominant gene encoding the major component of this mucus layer. The observed upregulation of *Muc5ac*, coupled with increased mucus thickness and enhanced goblet cell activity, may indicate a coordinated enhancement of overall mucus synthesis [43]. Upregulation of goblet cell–derived mucosal protective factors, such as *Tff1* and *Tff3*, contributes to the repair of epithelial tissues and the maintenance of microbial homeostasis [44]. These findings highlight the potential of coated FA in promoting host–microbiota symbiosis through mucosal reinforcement. Concurrently, the thickened mucus layer and elevated *Muc2* expression enhance the physical barrier against microbial translocation, thereby reducing epithelial contact and suppressing the release of pro-inflammatory cytokines, including TNF-α and IL-1β [45]. Our results consistently demonstrated that coated FA significantly upregulated *Muc2* expression and stimulated mucus secretion, leading to reduced adhesion and colonization of *C. rodentium*. In addition, coated FA exerted multifaceted protective effects by strengthening mucus barrier integrity (*Muc2, Muc5ac*), promoting mucosal repair (*Tff1, Tff3*), and attenuating intestinal inflammation, thereby suppressing both colonization and virulence of *C. rodentium*. These synergistic effects suggest that coated FA supports intestinal homeostasis via redox-driven modulation of the mucus-immune-microbiota interface.

Concomitantly, 16S rRNA sequencing revealed that coated FA treatment induced a beneficial shift in gut microbial composition, characterized by increased abundance of *Lactobacillus*, *Limosilactobacillus*, *Romboutsia*, and *Akkermansia* and decreased abundance of Enterobacteriaceae. In particular, *Akkermansia muciniphila* utilizes mucins in a non-destructive manner to stimulate mucus renewal, while *Limosilactobacillus reuteri* and *Romboutsia* exert immunomodulatory and biofilm-disrupting effects [46,47]. The observed reduction in Enterobacteriaceae likely reflects both direct ecological competition and an unfavorable redox environment, as this family preferentially expands under oxidative and inflammatory conditions [48]. These results further support the concept of the redox-mucus-microbiota axis, whereby antioxidant-mediated redox modulation rebuilds the gut ecosystem to enhance colonization resistance. To delineate the microbial functional mechanisms involved, the bacterial pathways that potentially influence mucus homeostasis were explored subsequently. Enterobacteriaceae, usually enriched under pro-oxidative conditta-host crosstalk, our results demonstrated that coated FA treatment established a protective mucosal environment through the modulation of key bacterial populations and their associated signaling pathways. It's worth noting that the abundance of *L. reuteri* was significantly increased following coated FA intervention, which was correlated with a marked upregulation of *Muc2* expression. The increased mucin production not only strengthened the physical mucus barrier but also attenuated IL-6-mediated inflammatory responses. Consequently, downstream activation of the iron-regulatory gene Fur and its targets (*FecB/FepB*) was diminished, thereby limiting iron availability for pathogenic Enterobacteriaceae. Furthermore, elevated *Muc2* levels activated *CsgD*, a central transcriptional regulator that promotes the expression of *PgaA/PgaB* and *BcsA*, thereby facilitating biofilm maturation and extracellular matrix formation-mechanisms that further enhance colonization resistance. On the contrary, the potentially pathogenic genus *Romboutsia*, which was frequently linked to oxidative stress and epithelial disruption, was markedly suppressed. This suppression resulted in decreased expression of *CsrB/CsrC* and *CsgA/CsgD*, leading to reduced epithelial adhesion and *Fur*-dependent iron uptake (*FhuB/FhuC*). Collectively, these findings underscore the role of coated FA-induced microbial remodeling in reinforcing mucosal defense mechanisms and curbing the pathogenic potential of Enterobacteriaceae. Further *in vitro* co-culture trial indicated that under a lower redox system, the pathogenic potential of *C*. *rodentium* were significantly inhibited. *L*. *reuteri* had been reported to improve barrier integrity, produce antimicrobial metabolites, and compete with pathogens for ecological niches. In particular *L*. *reuteri* exert immunomodulatory and biofilm-disrupting effects [49,50]. As a commensal colonizer, *L. reuteri* can occupy mucosal and nutritional niches in the gut, thereby reducing the available space for pathogen adherence including *C*. *rodentium* [51]. Moreover, the virulence of *C*. *rodentium* primarily depends on the locus of enterocyte effacement (LEE) pathogenicity island, with *ler* and *tir* as the key regulators [52]. Previous studies have shown that cell-free supernatants from *L*. *acidophilus* reduce autoinducer-2 levels in *Escherichia coli*, thereby significantly downregulating LEE-associated genes including *tir* and *ler* [53]. Our findings demonstrated that under lower redox potential, *L. reuteri* engaged in direct crosstalk with intestinal pathogens, and that the pathogenic activity of *C*. *rodentium* may vary depending on the intestinal microenvironment. In addition, probiotics may modulate the *ler-tir* regulatory axis of pathogens, inhibiting LEE gene transcription and attenuating virulence at the molecular level, consistent with the results observed in animal experiments.

## 5 Conclusion

In summary, coated antioxidants maintained high efficiency in redox potential reduction, effectively reversed the weight loss caused by pathogenic bacterial infections. Following *C. rodentium* infection, the coated antioxidant significantly altered the microbial community structure, consistently increased the abundance of *L. reuteri* while reducing the load of Enterobacteriaceae. By sustaining lower intestinal redox potential levels, coated FA further attenuated enterobacterial adhesion capabilities through enhanced biofilm formation regulation, maintained the integrity of the mucus barrier, and reducing *ler* and *tir* expression. These findings provide mechanistic insights into redox mediated microbial interactions and suggest a controlled release potential strategy for inhibition of pathogen colonization through sustain lower redox potential.

## CRediT authorship contribution statement

**Ni Feng:** Data curation, Formal analysis, Writing – original draft, Writing – review & editing. **Changsong Fu:** Data curation, Resources, Validation, Writing – original draft. **Jinwei You:** Formal analysis, Investigation, Validation. **Dongfang Wang:** Investigation. **Xiaobo Feng:** Conceptualization, Supervision, Writing – review & editing. **Yong Su:** Conceptualization, Funding acquisition, Supervision, Writing – review & editing.

## Declaration of competing interest

The authors declare that they have no competing financial interests or personal relationships that could have appeared to influence in this study.

## Data Availability

Data will be made available on request.
